# Relative Efficacy of Conventional Monotherapies and Select Nonconventional, Over‐the‐Counter Products for Male Androgenetic Alopecia: A Network Meta‐Analysis Study

**DOI:** 10.1111/jocd.70483

**Published:** 2025-10-06

**Authors:** Aditya K. Gupta, Mary A. Bamimore, Mesbah Talukder

**Affiliations:** ^1^ Mediprobe Research Inc. London Ontario Canada; ^2^ Division of Dermatology, Department of Medicine University of Toronto School of Medicine Toronto Ontario Canada; ^3^ School of Pharmacy BRAC University Dhaka Bangladesh

## Abstract

**Background:**

Society values a full head of hair. Therefore, androgenetic hair loss (AGA), though medically benign, can cause significant emotional distress. There is strong demand for alternative (nonconventional, over‐the‐counter) AGA treatments. It is important to have evidence on the efficacy of these treatments for AGA—especially in comparison with treatments that are approved by the United States Food and Drug Administration (FDA), such as oral finasteride and topical minoxidil.

**Aims:**

Following a systematic review, we conducted a network meta‐analysis (NMA) to determine the relative efficacy of conventional monotherapies and selected alternative (nonconventional, over‐the‐counter) products for male AGA.

**Methods:**

We conducted a Bayesian NMA under a fixed effect model with uniform priors; the NMA estimated relative effects—as per mean difference (MD), along with the 95% credible interval (CI)—and surface under the cumulative ranking curve (SUCRA) values. We also assessed study‐level evidence quality. Eligible studies were identified through systematic searches (without date restrictions) in PubMed and Scopus on April 30, 2025. The main outcome measure was change in total hair density at 24 weeks from baseline (in hairs/cm^2^).

**Results and Conclusion:**

We found 24 eligible trials—where the relative efficacy of eight conventional monotherapies and seven alternative (nonconventional, over‐the‐counter) products was determined. The current NMA study confirms the efficacy of conventional monotherapies such as oral dutasteride, topical/oral minoxidil, and oral/topical finasteride for male AGA. We have provided guidance regarding the relative efficacy of some alternative (nonconventional, over‐the‐counter) agents (e.g., melatonin (topical) and rosemary oil (topical)) compared to conventional treatments.

## 1 Introduction

Male androgenetic alopecia (male AGA) is characterized by progressive nonscarring hair loss caused by “hair follicle miniaturization” whose etiological origin can be either androgen‐dependent or androgen‐independent [1]. Furthermore, the basis of male AGA is also genetic with a polygenic mode of inheritance: [2] different mutations in the androgen receptor (AR) translate to various phenotypic manifestations of the pattern baldness [1]. Synonyms for male AGA include “male pattern hair loss” (MPHL) [3]. Though male AGA is clinically benign, it can be a direct cause of emotional distress because having a “full head of hair” is often associated with youthfulness and handsome looks, both cross‐culturally—and even cross‐generationally [4]. The established negative psychological consequences of male AGA [5, 6], the most prevalent form of alopecia, explain the ever‐high demand for hair loss therapies, which itself is a multi‐billion dollar industry [4, 7, 8].

Oral finasteride and topical minoxidil are long‐standing therapeutic agents approved by the United States (US) Food and Drug Administration (FDA) for the treatment of AGA [9]. The therapeutic action of finasteride—a 5‐alpha reductase inhibitor (5‐ARI)—is androgen‐dependent, while minoxidil's medicinal impact is presumed to operate predominantly independently of androgen metabolism [10]. The FDA approved oral finasteride 1 mg (mg) daily as a prescription treatment for male AGA in 1997 [11]; the FDA approved topical minoxidil 2% solution as a prescription medication for male AGA in 1988 [9, 10]. In 1996, topical minoxidil 2% solution became FDA‐approved for over‐the‐counter (OTC) use in male AGA [9]. In 1997, the FDA approved OTC use of topical minoxidil 5% solution for male AGA [9, 10]; topical minoxidil 5% foam became FDA‐approved for men with AGA in 2006 [9, 10].

In the United States alone, 50 million men have AGA [12]. Given the millions of individuals who are diagnosed with male AGA, in addition to how detrimental the condition is to quality of life, it is unsurprising that Castro et al. (2023) reported the market for hair loss therapies to be valued at $7.6 billion—with a projected market value of $13 billion by 2028 [12]. Alternative medicines for male AGA—which include nutraceuticals—hold a notable share of the multi‐billion dollar industry [4, 7, 8].

There is a high demand for alternative therapies for male AGA, as evidenced by its billion‐dollar market value. This demand can be explained by the fact that many consumers of hair loss therapies are laypeople who may not have the background to differentiate between gimmicks and sound science. Second, the increased transparency in adverse event reporting with the use of conventional medicines has increased concerns about the safety of some of these products while decreasing the layperson's apprehension toward alternative agents that are often marketed as void of untoward effects. Third, consumption of alternative options can be “positively reinforced” by the placebo effect.

Over the past decade, the male AGA literature has been expanded with published clinical trials on alternative, nonconventional, over‐the‐counter products (including nutraceuticals) [8, 13]. Though the publication of such trials has enriched the evidence base for alternative agents, it has—on the other hand—consequently introduced knowledge gaps in the comparative effectiveness of treatment options used by persons with male AGA. Hence, the aim of the current study was to, through network meta‐analysis (NMA), determine the relative efficacy of conventional monotherapies versus select alternative (nonconventional, over‐the‐counter) products for male AGA whose efficacy has been reported through the conduct of randomized controlled trials.

## 2 Materials and Methods

The protocol for our entire work was registered in the *International Platform of Registered Systematic Review and Meta‐analysis Protocols* (INPLASY) under the ID 202560102 [14]; furthermore, the conduct of our work followed the *Preferred Reporting Items for Systematic reviews and Meta‐Analyses* (PRISMA) guidelines for NMAs [15]. Given the voluminous options of nutraceuticals for AGA, we deemed it highly “pragmatic” to select 10 or fewer alternative (nonconventional, over‐the‐counter) agents for our NMA; our selection was guided by various published reviews on alternative options for treating male AGA, including the reviews by Ring et al. (2022) [8] by Rubaian et al. (2024) [13].

### 2.1 Searches

The peer‐reviewed literature was systematically searched in Scopus and PubMed on April 30, 2025, to obtain data from studies that met the following criteria as per the PICO (patient, intervention/comparator, outcome) framework: eligible studies had to be published in English and had to have an arm that investigated the impact of a conventional monotherapy, or an alternative (nonconventional, over‐the‐counter) product, on the 24‐week change in total hair density (in hairs/cm^2^) across persons with AGA. We used the *Systematic Review Accelerator* deduplicator web tool [16] to remove duplicate records. The two stages of screening of titles/abstracts and full text were performed independently by two authors (MT and MAB). Any discrepancy throughout the search process was resolved through discussion with a third author (AKG).

### 2.2 Evidence Quality

Trials that met our eligibility criteria were assessed for evidence quality with the *Risk‐of‐bias VISualization* (robvis) tool [17]. The tool graphically summarizes study‐level evidence quality with “traffic plots.”

### 2.3 Network Plot

We produced a network plot for the current study's outcome of interest; a network plot is a graph of “nodes” and “edges.” A node corresponds to an intervention (or comparator) of interest—and also represents the level (or unit) of analysis; nodes are represented as vertices, and an edge is depicted as the line between two vertices and represents the direct comparison of two interventions from an actual head‐to‐head trial.

### 2.4 Inconsistency and Transitivity

A network meta‐analysis estimates the relative effect of interventions that have not actually been compared in a head‐to‐head trial—and such estimates represent “indirect evidence.” Hence, for a network's estimates to be valid, it must be “transitive”—where consistency is a “statistical manifestation” of transitivity. Node‐splitting analysis for inconsistency determines whether direct evidence agrees with indirect evidence, such that the null hypothesis of no inconsistency is rejected when *p* values are significant (i.e., *p* < 0.05) [18].

### 2.5 Network Meta‐Analysis

We conducted a Bayesian NMA under a fixed‐effects model with uniform priors. An NMA estimates interventions' relative efficacy and Surface Under the Cumulative Ranking curve (SUCRA) values. A comparator's SUCRA value ranges from 0 to 1 (or 0%–100%) and serves as a “rank metric” for efficacy; NMAs estimate relative effects of comparators for every possible combination in a pairwise manner. For relative efficacy, our point estimate was the mean difference (MD); 95% credible interval (CI) is also provided for each MD. The threshold for statistical significance was 5% (or 0.05). We used a Kilim plot [19] to present SUCRA values of comparators.

### 2.6 Sensitivity Analysis

We conducted a sensitivity analysis where we adjusted for variation in disease severity.

## 3 Results

**FIGURE 1:**
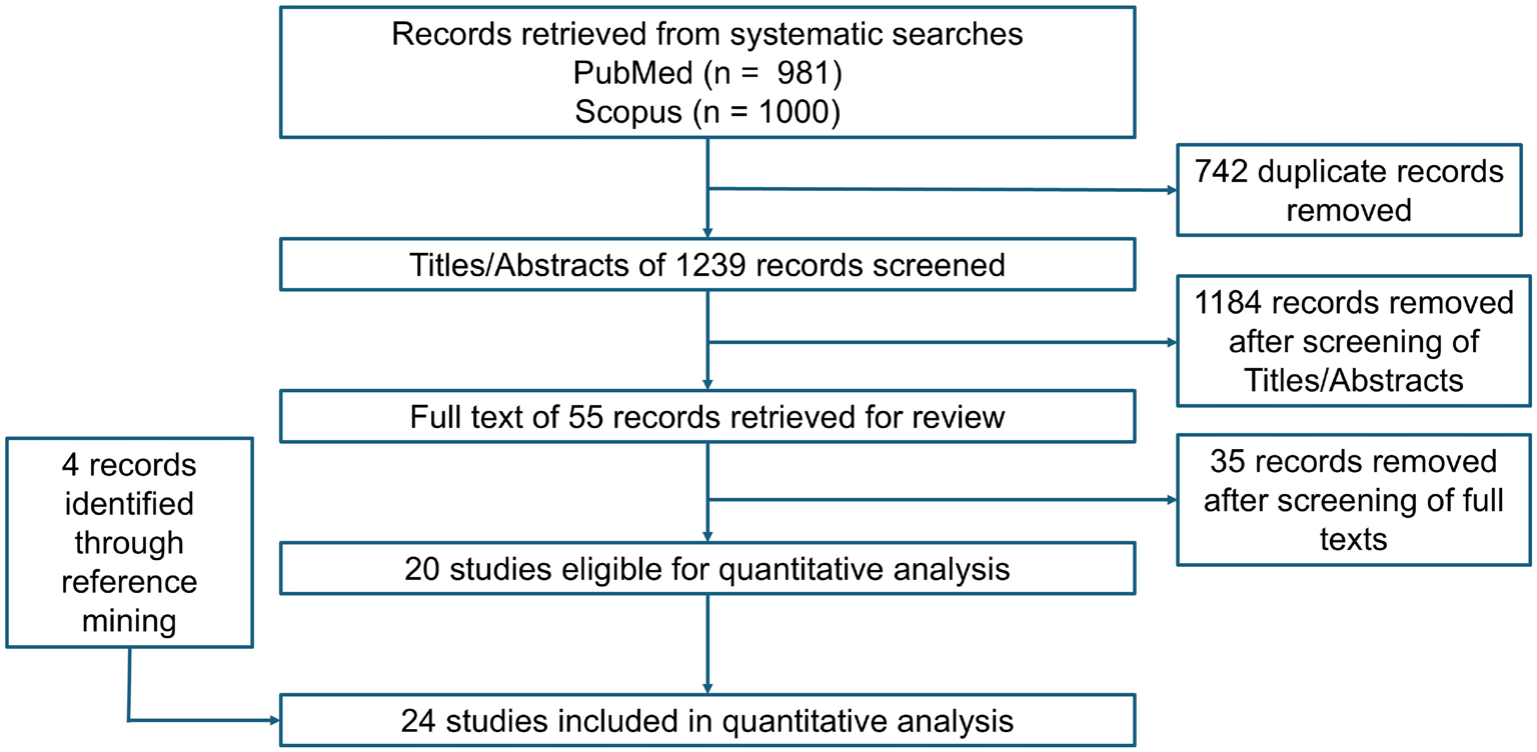
Schematic for the identification of eligible studies. This flow chart summarizes the search process for the identification of eligible studies.

**FIGURE 2:**
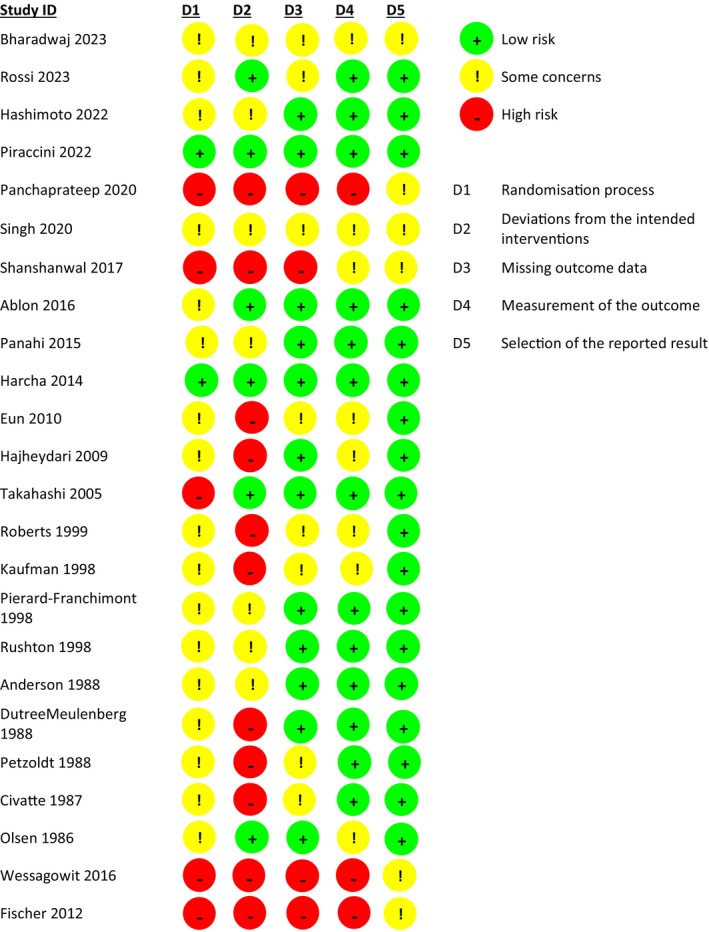
Qualitative summary for included studies' risk of bias. In this traffic plot, green, yellow, and red correspond to low, moderate, and high risk of bias, respectively.

**FIGURE 3:**
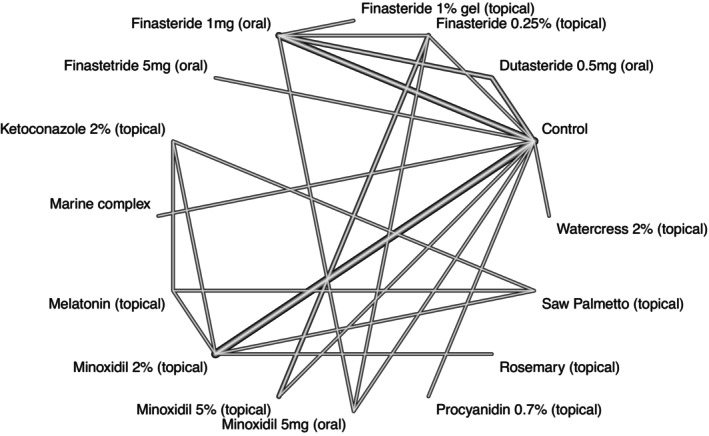
Network plot. This graph represents the connection of comparators across our network for a 24‐week change in total hair density. The thickness of the line indicates the number of studies. The thicker the line, the greater the number of studies.

We identified 25 studies whose data were used for our NMA—and the identification process for the eligible studies is summarized in Figure 1; an arm‐level summary of the eligible studies' characteristics is presented in Table 1 [20, 21, 22, 23, 24, 25, 26, 27, 28, 29, 30, 31, 32, 33, 34, 35, 36, 37, 38, 39, 40, 41, 42, 43]. Our study‐level evaluation of evidence quality is provided in the traffic plot in Figure 2. The network plot for our outcome of interest is depicted in Figure 3; results from our node‐splitting analysis for inconsistency are presented in Table 2. Node‐splitting analysis for inconsistency could not be performed for some comparisons because of the geometry of the network (Figure 3) [44].

**TABLE 1:** Summary of included studies' arm‐level characteristics [20, 21, 22, 23, 24, 25, 26, 27, 28, 29, 30, 31, 32, 33, 34, 35, 36, 37, 38, 39, 40, 41, 42, 43].

Study (first author, year)	Intervention	Frequency	Sample size at baseline (n)	Age ± SD (years)^a^
Bharadwaj 2023 [20]	Finasteride 0.25% (topical)	Twice daily	20	27.1 (10–36)
	Minoxidil 5% (topical)	Twice daily	20	25.6 (18–37)
Rossi 2023 [21]	Minoxidil 5% (topical)	Twice daily	11	23.5 ± 2.2
	Finasteride 0.25% (topical)	Once daily	12	23.7 ± 2.2
Hashimoto 2022 [22]	Watercress 2% (topical)	Twice daily	23	20 to 55
	Control	Twice daily	23	20 to 55
Piraccini 2022 [23]	Finasteride 0.25% (topical)	Once daily	189	32.5 ± 5.4
	Finasteride 1 mg (oral)	Once daily	85	32.3 ± 5.5
	Control	Once daily	184	31.8 ± 4.9
Panchaprateep 2020 [24]	Minoxidil 5 mg (oral)	Once daily	30	38 ± 10
Singh 2020 [25]	Minoxidil 5% (topical)	Twice daily	20	18–60
	Control	Twice daily	20	18–60
Shanshanwal 2017 [26]	Dutasteride 0.5 mg (oral)	Once daily	45	18–40
	Finasteride 1 mg (oral)	Once daily	45	18–40
Ablon 2016 [27]	Marine complex	Once daily	30	42.8 ± 7.7
	Control	Once daily	30	46.1 ± 7.6
Panahi 2015 [28]	Rosemary (topical)	Twice daily	50	24.78 ± 3.67
	Minoxidil 2% (topical)	Twice daily	50	23.38 ± 2.5
Harcha 2014 [29]	Dutasteride 0.5 mg (oral)	Once daily	184	38.6 ± 7.66
	Finasteride 1 mg (oral)	Once daily	179	38 ± 7.81
	Control	Once daily	181	38.7 ± 8.43
Eun 2010 [30]	Dutasteride 0.5 mg (oral)	Once daily	73	37.8 ± 7.1
	Control	Once daily	75	38.4 ± 6.6
Hajheydari 2009 [31]	Finasteride 1 mg (oral)	Once daily	19	22.8 ± 3.3
	Finasteride 1% gel (topical)	Twice daily	19	22.8 ± 3.3
Takahashi 2005 [32]	Procyanidin 0.7% (topical)	Twice daily	25	27 to 58
	Control	Twice daily	24	27 to 58
Roberts 1999 [33]	Finasteride 1 mg (oral)	Once daily	117	30 ± 0.4
	Finasteride 5 mg (oral)	Once daily	111	30 ± 0.4
	Control	Once daily	116	30 ± 0.3
Kaufman 1998 [34]	Finasteride 1 mg (oral)	Once daily	779	32 ± 6.82
	Control	Once daily	774	32.5 ± 6.98
Pierard‐Franchimont 1998 [35]	Minoxidil 2% (topical)	2–4 times weekly	4	21–33
	Ketoconazole 2% (topical)	2 to 4 times weekly	4	21–33
Rushton 1998 [36]	Minoxidil 2% (topical)	Twice daily	12	18–49
	Control	Twice daily	12	18–49
Anderson 1988 [37]	Minoxidil 2% (topical)	Twice daily	77	32.8 (19–49)
	Control	Twice daily	77	32.8 (19–49)
DutreeMeulenberg 1988 [38]	Minoxidil 2% (topical)	Twice daily	74	34.3 (19–49)^b^
	Control	Twice daily	70	
Petzoldt 1988 [39]	Minoxidil 2% (topical)	Twice daily	101	32.9 (17–49)^b^
	Control	Twice daily	100	
Civatte 1987 [40]	Minoxidil 2% (topical)	Twice daily	125	33.4 (18–49)
	Control	Twice daily	122	34.8 (20–40)
Olsen 1986 [41]	Minoxidil 2% (topical)	Twice daily	19	35.4 ± 4
	Control	Twice daily	19	37 ± 6.3
Wessagowit 2016 [42]	Saw Palmetto (topical)	Once daily	100	55.12 ± 6.25
Fischer 2012 [43]	Melatonin (topical)	Once daily	35	18–41

Abbreviations: mg, milligram; SD, standard deviation.

^a^ The range was reported along with the mean, age, as the arm‐level standard deviation was not provided.

^b^ Arm level information was not provided; mean age and range were reported across all participants.

**TABLE 2:** Results from node splitting analysis for assessment of inconsistency.

Comparison	*p*	Effect size (95% credible interval)
Control, Minoxidil 5% (topical)	0.41695	
Direct		34.0 (−2.9, 72.0)
Indirect		17.0 (−3.0, 40.0)
Network		20.0 (3.7, 41.0)
Control, Dutasteride 0.5 mg (oral)	0.024725	
Direct		15.0 (5.20, 22.0)
Indirect		34.0 (20.0, 48.0)
Network		19.0 (9.5, 28.0)
Control, Finasteride 0.25% (topical)	0.470275	
Direct		14.0 (−4.60, 33.0)
Indirect		30.0 (−11.0, 70.0)
Network		15.0 (0.85, 30.0)
Control, Finasteride 1 mg (oral)	0.002675	
Direct		13.0 (10.0, 18.0)
Indirect		−11.0 (−23.0, 0.77)
Network		12.0 (4.20, 19.0)
Minoxidil 5% (topical), Finasteride 0.25% (topical)	0.423675	
Direct		−3.5 (−19.0, 8.8)
Indirect		−21.0 (−60.0, 9.0)
Network		−4.90 (−21.0, 6.50)
Dutasteride 0.5 mg (oral), Finasteride 1 mg (oral)	0.0449	
Direct		−12. (−22, −2.6)
Indirect		7.4 (−8.9, 24.)
Network		−7.4 (−16, 2.0)
Finasteride 0.25% (topical), Finasteride 1 mg (oral)	0.417675	
Direct		−0.91 (−19.0, 18.0)
Indirect		−19.0 (−59.0, 23.0)
Network		−3.7 (−19.0, 11.0)

*Note:* For a given comparison, a *p* value of 0.05 or above corresponds to no significant inconsistency, which suggests that direct and indirect evidence are in agreement.

Abbreviation: mg, milligram.

**FIGURE 4:**
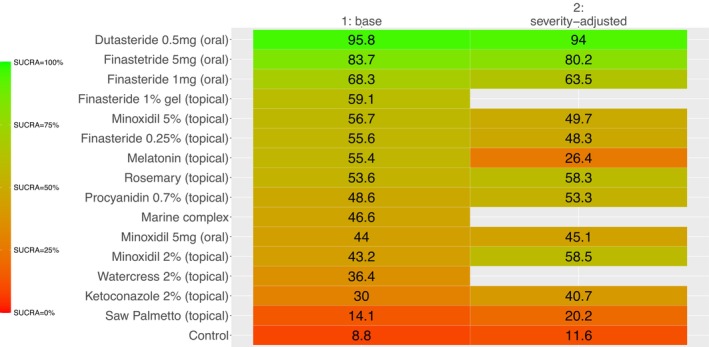
Kilim plot for interventions' relative efficacy. A comparator's surface under the cumulative ranking curve (SUCRA) values reflect its overall rank for efficacy, where higher values indicate greater (i.e., favorable) impact. The vertical axis corresponds to the comparators; our network consisted of 19 active comparators and a control node that amalgamated placebo and vehicle. The horizontal axis represents endpoints, where the leftmost, middle, and rightmost columns represent SUCRA values from the base and severity‐adjusted network meta‐analyses, respectively. The SUCRA values in the respective columns are in percentages. A color chart illustrating SUCRA values is provided herein; the colors red and green (chosen arbitrarily) represent the lowest and highest SUCRA values, respectively. This, in turn, signifies the treatments ranked from least to most effective according to this metric.

Comparators' SUCRA values from the base and sensitivity analyses are presented in the Kilim plot in Figure 4.

Through the systematic searches, we identified eight conventional monotherapies; our review of the literature guided us to seven alternative (nonconventional, over‐the‐counter) products. Across the 24 trials (Table 1), we determined the relative efficacy of eight conventional monotherapies, namely, dutasteride 0.5 mg (oral), finasteride 0.25% (topical), finasteride 1% gel (topical), finasteride 1 mg (oral), finasteride 5 mg (oral), minoxidil 2% (topical), minoxidil 5% (topical), minoxidil 5 mg (oral), and the nonconventional therapies, namely, ketoconazole 2% (topical), marine complex, procyanidin 0.7% (topical), rosemary (topical), melatonin (topical), saw palmetto (topical), and watercress 2% (topical).

Each comparator's frequency of use is detailed in Table 1. Our node‐splitting analysis showed agreement between direct and indirect evidence across our network, as the *p* value for the majority of the comparisons was above 0.05 (Table 2).

The most efficacious comparator was dutasteride 0.5 mg (oral) in the base NMA (SUCRA = 95.8%) and severity‐adjusted NMA (SUCRA = 94%).

## 4 Discussion

The FDA, the European Union Medicines Authority (EMA), the UK Medicines and Health Care Products Regulatory Agency, and Health Canada have issued alerts regarding possible associations between oral/topical finasteride, such as psychiatric adverse events and sexual dysfunction, sometimes persisting after product discontinuation [45]. Thus, there has been a desire to look for alternative (nonconventional, over‐the‐counter) options that may be effective and safe for managing AGA.

The current NMA study addresses some knowledge gaps on the comparative impact of alternative (nonconventional, over‐the‐counter) products and conventional monotherapies for male AGA. To our knowledge, the relative efficacies of conventional monotherapies and alternative (nonconventional, over‐the‐counter) agents for male AGA have not been previously reported. Through a systematic review of the literature, we selected seven nonconventional, over‐the‐counter “alternative” products whose efficacy for male AGA has been confirmed through the conduct of randomized controlled trials. Beyond the nonconventional OTC products, several nontraditional, device‐based or mechanotherapy interventions, such as low‐level laser therapy (LLLT), other electromagnetic‐based modalities (e.g., pulsed electromagnetic fields, radiofrequency), and scalp massage, have shown signs of efficacy for MPHL in small randomized or controlled studies and pilot trials [46, 47, 48]. These interventions were excluded from this NMA because they focus exclusively on nonconventional OTC products.

The mode of action of many of these alternative options has not been as well studied as the conventional monotherapies. For example, saw palmetto may inhibit the 5‐alpha reductase enzyme [49], procyadin may reduce oxidative stress and inflammation [49, 50], and rosemary oil is thought to act via different mechanisms (anti‐oxidative, anti‐inflammatory, and antifungal) [28, 49].

The response to topical minoxidil can be variable and is in part dependent on genetic factors such as the activity of the sulfotransferase 1A1 (SULT1A1) enzyme [51]. This converts minoxidil (a pro‐drug) to its active form, minoxidil sulfate. Individuals with low SULT1A1 activity are more likely to be poor responders compared to those with high SULT1A1 activity [51, 52, 53]. In most clinical trials evaluating the efficacy of topical minoxidil, the levels of SULT1A1 are not quantified. A study reported that 75% of individuals who used the SULT1A1 adjuvant in combination with daily 5% topical minoxidil for 60 days experienced hair regrowth, compared to just 33% of those who received 5% topical minoxidil plus a placebo adjuvant (*p* = 0.023) [51]. It is therefore likely that the true efficacy of topical minoxidil has been underestimated in trials where the levels of SULT1A1 were not quantified.

The congruence between direct and indirect evidence in our node‐splitting analysis and congruent estimated pairwise relative effects across our base and sensitivity analyses substantiates the robustness of our Bayesian NMA. Furthermore, the strength of our comparative analysis is buttressed by the fact that the current study's findings tightly resonate with those of recent NMA studies [54, 55].

Another strength of our NMA study is that our outcome measure did not amalgamate the efficacy of treatment at different time points (e.g., we have reviewed the efficacy at 6 months from the start of treatment and did not collapse the efficacy metrics at 3, 4, or 9 months into one measure). Our NMA did not include nonconventional treatments such as rice bran extract and pumpkin seed oil, to name a few. For example, we did not use efficacy data for pumpkin seed oil from the study by Ibrahim et al. (2021) [56] because the total hair density was not measured at 24 weeks; similarly, rice bran extract, whose efficacy was estimated in the trial by Choi et al. (2015) [57], was not among our comparators as total hair density was not measured at 24 weeks.

We also adhered to the exact outcome measure (terminal hair/cm^2^ at week 24) and did not include studies that used alternate outcome measures, for example, photographic evidence.

A limitation of the current work is that variation in participants' disease duration, at baseline, was not accounted for in our analyses—because of lack of data availability. Notwithstanding that, we adjusted for severity, as a sensitivity analysis, to indirectly address duration, as disease duration and severity may have a collinear relationship.

Our work has clinical and research implications. For male AGA, our results allow for evidence‐based conversations on conventional monotherapies and alternative (nonconventional, over‐the‐counter) products among stakeholders—including consumers, patients, and clinicians. Furthermore, the conduct of more head‐to‐head trials would permit the determination of relative efficacy at longer time points beyond week 24. Additionally, many of the RCTs evaluating alternative agents for male AGA have been conducted in a relatively smaller number of subjects compared to the larger trials evaluating the efficacy of conventional monotherapies.

Be they fad or fact, alternative (nonconventional, over‐the‐counter) interventions, including nutraceuticals, are here to stay. From time immemorial, human culture has always put a premium on voluminous cranial hair, and this explains why the oldest complete medical text known to man (i.e., the Ebers Papyrus from 1500 bc) inscribed remedies for various dermatologic conditions, including alopecia [58].

Male AGA has always been indifferent to socioeconomic class, as notable historical figures from King Louis XIII of France to King Charles II of England to the Greek physician Hippocrates all grappled with AGA. Interestingly, numerous pharmacopeias document how lay people and aristocrats (including Hippocrates) produced and consumed what modern medicine today calls nonconventional “snake oil medicine.”

Our NMA study has occurred at an opportune moment, given that well‐conducted randomized, controlled trials on alternative (nonconventional, over‐the‐counter) products are now available in the peer‐reviewed literature. Moreover, media‐ and market‐driven consumptions of alternative (nonconventional, over‐the‐counter) options arguably put an onus on the medical community to “scientize” this fad as much as possible, for the greater good.

## Author Contributions

Conception of the manuscript was done by A.K.G. Data analysis was performed by M.A.B. The manuscript was drafted by A.K.G., M.T., and M.A.B., substantively edited, and revised by A.K.G., M.T., and M.A.B.

## Ethics Statement

Approval from an ethics board was not required as there was no direct involvement with human participants.

## Consent

The research did not involve direct interaction with human participants; therefore, informed consent was not required.

## Conflicts of Interest

The authors declare no conflicts of interest.

## Data Availability

Data can be made available upon reasonable request.
